# COVID-19 pediatric vaccine authorization, FDA authority, and individual misperception of risk

**DOI:** 10.1093/jlb/lsae006

**Published:** 2024-04-19

**Authors:** Joanna K Sax, Neal Doran

**Affiliations:** California Western School of Law, 225 Cedar St., San Diego, CA 92101, USA; University of California San Diego, 9500 Gilman Drive, La Jolla, CA 92093, USA

**Keywords:** COVID-19 vaccine, vaccine hesitancy, notice and comment, intervention, Food and Drug Administration, public health

## Abstract

Vaccines are one component to the public health strategies to alleviate the COVID-19 pandemic. Hesitancy regarding COVID-19 vaccines in the United States has been problematic, which is not surprising given increasing overall vaccine hesitancy in recent decades. Most vaccines are administered during childhood years. Consequently, understanding hesitancy toward administration of vaccines in this age group may provide insight into possible interventions to reduce vaccine hesitancy. The present study analyzed a subset of over 130,000 public comments posted in response to a notice of meeting of the vaccine advisory group to the Food and Drug Administration. The meeting addressed whether to recommend Emergency Use Authorization (‘EUA’) of the COVID-19 vaccine for children ages 5–11. The results of the study demonstrate that most comments opposed EUA and these comments were associated with statements that indicated misperceptions of risk. Findings provide interesting insights regarding the role of public comments generally but also suggest that the public participation process in notice and comment can be modified to serve as an intervention to align individual perceptions of risk more closely with evidence-based assessment of risk. In addition, the findings provide opportunities to consider strategies for public health messaging.

## I. INTRODUCTION

The public health recommendations surrounding the COVID-19 pandemic generated enormous controversy across the United States, with the COVID-19 vaccine emergency use authorization as no exception. While some individuals waited anxiously for vaccines to be available, others took a wait-and-see approach, and still others refused to be vaccinated—all with variations in between.^1^ This continuum existed for the adults and was entirely predictable when it came to authorization for the COVID-19 vaccine for children.

Understanding both vaccine hesitancy and vaccine refusal can provide valuable insight to engage with the public to attempt to close the divide between individual perceptions and evidence-based assessments of risk.^2^ When the Food and Drug Administration (‘FDA’) authorizes a vaccine, it does so based on the evidence-based assessment of risk and a risk–benefit ratio.^3^ Vaccine hesitancy and vaccine refusal are based, at least in part, on misperceptions of risk.^4^

Risk perceptions are studied as part of individual decision-making. Decision-making is complicated and several theories exist to help us understand how individuals assign risk.^5^ Two theories with empirical support include Ambiguity Aversion and Affect Heuristic. Ambiguity Aversion posits that people are sensitive to lack of clarity about risk. Specifically, if individuals receive missing or conflicting information, they may assign a high risk and low benefit, regardless of the evidence-based assessment of risk.^6^ The Affect Heuristic centers on the hypothesis that decision-making is modulated by negative emotions. For example, if an individual experiences fear or dread when evaluating risk of a particular action, they are more likely to assign a high risk and low benefit to that action, regardless of the evidence-based assessment of risk.^7^

Risk regulation is a part of legal policy creation and implementation.^8^ Increasingly, individuals are challenging legal policies based on their individual perceptions of risk. In the COVID-19 pandemic, there have been legal challenges to mask mandates, restrictions on gatherings, and other policies aimed to reduce the spread of the virus that causes COVID-19.^9^ As the vaccine(s) aimed at the virus that causes COVID-19 disease entered the stage after challenges to non-pharmaceutical interventions, resistance to the vaccines was predictable. In addition, vaccine hesitancy in general had been an increasing problem even prior to the pandemic.^10^

On the regulatory eve of deciding Emergency Use Authorization (‘EUA’) for the Pfizer COVID-19 vaccine for children ages 5–11, the FDA issued a request for comments on its public docket.^11^ Approximately 130,000 comments were made, which is an enormous number of comments on a public docket.^12^ The present study sought to utilize the Ambiguity Aversion and Affect Heuristic theories to evaluate whether perception of risk might help explain opposition to the EUA of the COVID-19 vaccine for children ages 5–11.

The development and authorization of the COVID-19 vaccine for pediatric populations followed the development and authorization of the COVID-19 vaccine for adult populations. This became a bit of a double-edged story. In one sense, the FDA was familiar with the development of the COVID-19 vaccine for adult populations, especially as relevant here with the mRNA vaccine technology.^13^ Thus, the regulatory review for the pediatric population started against a backdrop of ongoing discussions about efficacy, safety, and risk from a regulatory perspective.^14^ At the same time, members of the population had raised specific risk perception concerns about the COVID-19 vaccines for adults; thus, those discussions were ongoing as well. Put differently, the EUA for the COVID-19 vaccine for pediatric populations occurred against a backdrop of EUA authorization of COVID-19 vaccines for the adult population.

The primary goal of the empirical study was to analyze whether any trends related to risk perception underscored why a commenter supported, opposed, or was neutral as to FDA EUA for the COVID-19 vaccine in children ages 5–11.^15^ We hypothesized that those commenters who were against EUA would be more likely to use affective words suggesting fear or dread.^16^ We also hypothesized that missing or conflicting information such as that the vaccine is experimental (missing information) or that the vaccine is more dangerous than the disease (conflicting information) might be associated with comments that opposed EUA.^17^ The results of the study suggest some trends that may prove fruitful for ways to close the divide between an individual’s perception of risk and evidence-based assessment of risk. This is important to tackle public health issues, as well as other areas in which the law seeks to regulate risk.

This article proceeds in four parts. Section II describes risk perception research that underscores the categories utilized in our study. The purpose of this section is to build on decades of research analyzing decision-making as it relates to risk perception and connect that research to COVID-19 vaccine hesitancy. Section III describes the administrative advisory committee and request for comments. The legal landscape for public participation through a regulatory process provides a window into how individuals receive and perceive legal interventions, in this case a meeting that advised the FDA to authorize a COVID-19 vaccine. Section IV describes an empirical study designed to understand whether comments made on the federal register are associated with risk perception theories. Section V discusses the legal implications of the empirical study. This section analyzes whether legal interventions can effectively respond to an individual’s perception of risk. Ultimately, this section posits that aligning an individual’s perception of risk with evidence-based assessment of risk is needed prior to the implementation of legal policy. Scholarship in risk regulation often focuses on the legal policy itself as the mechanism to handle risk or even the perception of risk. This article proposes that efforts and/or interventions aimed to align individual perception of risk with evidence-based assessment of risk should be addressed prior to the implementation of legal policy.

## II. RISK PERCEPTION

How an individual perceives risk is a complicated psychological phenomenon with cognitive, affective, and social components. Decades of research analyzing the underlying mechanisms demonstrates that multiple theories help us to understand risk perception. Not surprisingly, leaders in this area of research often collaborate and recognize the complicated nature of risk perception as part of decision-making.^18^ Two theories with empirical support are the Affect Heuristic and Ambiguity Aversion. These two theories, described below, form the basis for the risk perception categories in the empirical study described in Section IV.

### II.A. Affect Heuristic

The Affect Heuristic focuses on a ‘faint whisper of emotion’ called affect.^19^ This theory is pioneered by the work of Paul Slovic. As described by Slovic: ‘Affective responses occur rapidly and automatically – note how quickly you sense the feelings associated with the stimulus word “treasure” or the word “hate.” Reliance on such feelings can be characterized as “the affect heuristic.”’^20^ The affective component of decision-making is part of the quick-thinking process, termed Type 1.^21^ Type 1, also known as ‘fast thinking’, relies on heuristics, such as things that people can easily recall.^22^ In making decisions related to risk perception, affect offers cues.^23^

Empirical studies demonstrate support for the Affect Heuristic. A study by Alhakami and Slovic found that the relationship between risk and benefit was linked to the strength of the participants’ affect associated with the activity.^24^ In this study, the researchers found that rating activity on a binary scale such as good/bad, nice/awful, dreaded/not dreaded, etc., demonstrated that ‘people base their judgments of an activity or technology not only on what they *think* about it but also on how they *feel* about it.’^25^ How they ‘feel’ about it impacts their risk perception… if they feel positively, then they tend to perceive risk as low and benefit as high; if they feel negatively, then they tend to perceive risk as high and benefit as low.

Other studies show support that affect is a component of risk perception toward hazards or new technologies. Affect is an underlying predictor of risk perception to chemical items, nuclear power, and genetically engineered food, for example.^26^ Understanding how individuals perceive risk may provide insights into risk communication and legal strategies.^27^

Interestingly, affect has a layered application, including a problem of a numbing effect.^28^ That is, an individual might have an affective response at hearing about one person’s suffering or death but then have less of a response upon learning of the difference between 500 and 600 deaths.^29^ This type of insensitivity is referred to a ‘psychological numbing’ and one wonders whether this is applicable to the large number of deaths caused by COVID-19 and the lack of interest in public health measures suggesting numbness toward continued numbers of hundreds of deaths each day.^30^

As the law seeks to control social behaviors, understanding individual’s affective responses to technology or to novel interventions such as vaccines is imperative to obtain acceptance and compliance. As insightfully explained by Paul Slovic:

Ultimately, understanding the role of affect will inform age-old questions regarding the nature of human rationality. Contemplating the workings of the affect heuristic helps to appreciate Damasio’s contention that rationality is not only a product of the analytical mind, but of the experimental mind as well. The perception and integration of affective feelings, within the experimental system, appears to be the kind of high-level maximization process postulated by economic theories since the days of Jeremy Bentham. These feelings form the neural and psychological substrate of utility. In this sense, the affect heuristic enables us to be rational actors in many important situations. But not in all situations. It works beautifully under some circumstances and fails miserably in others. The law must learn to tell the difference.^31^

In sum, the affect heuristic is a component of risk perception that is intricately associated with legal interventions. Understanding the affective component to risk perception may provide valuable insight to the development of interventions that close the divide between evidence-based assessment of risk and individual’s perception of risk. Importantly, what is instructive about Slovic’s observations is that the ‘law must learn to tell the difference’ between when the affect heuristic works well and when it does not.^32^

### II.B. Ambiguity Aversion

Ambiguity aversion posits that individuals make decisions in the face of uncertainty and that this may lead to an inappropriate assignment of risk.^33^ The uncertainty is referred to as ambiguity, which is defined as ‘a quality depending on the amount, type, reliability, and “unanimity” of information, giving rise to one degree of “confidence” in an estimate of relative likelihoods.’^34^ In the face of conflicting information, decisions regarding probabilities become potentially less reliable because it may be difficult to rule out information.^35^ In other words, people make judgments in the face of missing or conflicting information and these judgments do not necessarily follow a pure economic model of rational decision-making.^36^ Decisions under ambiguous conditions may tend to prefer ‘known risks’ as compared with ‘unknown risk(s)’ of an innovation, even if the ‘unknown risk’ can be assigned to be a likely lower risk value as compared with the ‘known risk’.^37^ Ambiguity aversion is also described as ‘vagueness about outcome probabilities (typical in economics) as well as aversion to novelty, complexity and insolubility.’^38^

Technology, such as vaccines or genetically engineered food, provide examples of innovation in which individuals may experience ambiguity aversion due to what they consider ‘unknown risk(s).’ Blaisdell and colleagues analyzed perceived risks of vaccines in a group of Vaccine Hesitant Parents (‘VHPs’) and learned that the VHPs found ambiguity in information about the harms of vaccines ‘citing concerns about missing, conflicting, changing, or otherwise unreliable nature of information’.^39^ In this study, the VHPs perceived the risk of vaccination as greater than the risk of vaccine preventable disease: a perception that is not in line with evidence-based assessment of risk.^40^ In a different study, Sax and Doran reported that individuals who showed an initial aversion to ambiguous information were significantly more likely to indicate a high risk assessment to various areas of technology when presented with missing or conflicting information.^41^ These studies suggest that individuals perceive risk as higher with innovations. It is possible that exposure to both information from healthcare professionals regarding safety of vaccines, and misinformation regarding lack of safety of vaccines found on the internet may create conflicting information and contribute to vaccine hesitancy.

### II.C. Risk Perception and COVID-19 Vaccines

If the past is the best predictor of the future, then vaccine hesitancy and vaccine refusal toward the COVID-19 vaccines was to be expected.^42^ Over the past several decades, vaccine refusal and vaccine hesitancy grew even toward well-established vaccines, such as the those protecting against measles, mumps, and rubella.^43^ Outbreaks of measles in California, for example, prompted the California state government to remove a philosophical/religious vaccine exemption for school age children.^44^ Studies document the rise of vaccine hesitancy and refusal.^45^ Thus, especially, with the novel ability to capture mRNA technology for the COVID-19 vaccines, it was not surprising that vaccine hesitancy and refusal would grow over time. Given the novelty of the mRNA vaccine technology, this provides fodder for manipulating decision-making.^46^

While many adult Americans received the COVID-19 vaccine, a proliferation of vaccine hesitancy and vaccine refusal increased over time.^47^ The uptake of the COVID-19 vaccine among the adult population is much greater than the uptake among children.^48^ This suggests that the trend in VHPs extended toward administration of the COVID-19 vaccine to children. In July 2022, the Kaiser Family Foundation reported:

[R]eported vaccine uptake among children ages 5-11 has also slowed in recent months. Four in ten parents of kids ages 5-11 now report their child has gotten vaccinated (40%). Just 1% of parents now say they will get their child vaccinated right away, while about one in ten parents of 5-11 year-olds still want to “wait and see.” Notably, nearly half of parents of children ages 5-11 say they either will only get them vaccinated if required to do so (10%) or say they definitely won’t get their 5-11 year-old vaccinated (37%).^49^

This is compared to adults:

The latest COVID-19 Vaccine Monitor finds that around three-quarters of adults (76%) say they have gotten at least one dose of a COVID-19 vaccine, a share that continues to hold relatively steady since September 2021. This includes around half of adults who say are fully vaccinated and also received a COVID-19 booster dose (49%), a quarter who have been fully vaccinated but have not gotten their booster (24%), and a small share who are partially vaccinated (2%).^50^

These data demonstrate that despite wide availability of COVID-19 vaccines, many adults refrain from being fully vaccinated and/or boosted. The numbers for unvaccinated children are even higher.

When asked about their concerns regarding vaccinating children, responses included that parents were very or somewhat concerned about side effects, unknown long-term effects and lack of effectiveness.^51^ The VHPs appear to misperceive the risk of COVID-19, which is in line with other studies analyzing underlying reasons for vaccine hesitancy.^52^ Understanding the disconnect between the misperception of risk of the vaccine (and relatedly the misperception of risk of the vaccine preventable disease) and evidence-based risk assessment is important. Layered on top of that is the question of whether engaging with the public through the regulatory process could be an avenue to close the divide. The present study, described in Section IV below, sought to analyze public comments made on the Federal Register to better understand misperception of risk of the COVID-19 vaccine and to discuss whether engagement through the regulatory process could contribute to closing the divide.

The vaccines offering protection against COVID-19 entered the authorization process not only in the face of increasing vaccine hesitancy among the population in the United States (‘US’) but also with a new form of vaccine technology, the ability to harness and stabilize mRNA.^53^ This article focuses on the mRNA COVID-19 vaccines because the Pfizer mRNA COVID-19 vaccine was the first vaccine to go through regulatory authorization for children ages 5–11.

Understanding whether individuals inappropriately assign risk to the COVID-19 vaccines is important not only for risk perception as it relates to innovation generally, but specifically to attempt to mitigate the impact of the COVID-19 pandemic and provide lessons for the next pandemic. In addition, although secondarily, considering whether the public participation process through notice and comment on the Federal Register offers an opening for intervention(s) aiming to align individual perception of risk with evidence-based assessment of risk, can offer some indicia of the utility of the notice and comment process. The next section, Section III, provides an overview of the administrative framework as background to the empirical study, which is then presented in Section IV.

## III. ADMINISTRATIVE PROCEDURE REGARDING PUBLIC COMMENTS RELATED TO EUA OF THE COVID-19 VACCINE FOR CHILDREN AGES 5–11

### III.A. An Overview of the Regulatory Scheme for the Vaccines and Related Biological Products Advisory Committee

The Administrative Procedures Act of 1946, as amended, (‘APA’) governs the establishment and rules regarding agencies.^54^ The FDA and the Centers for Disease Control (‘CDC’) are two agencies intimately involved in vaccine authorization and recommendations.^55^ Agencies are allowed to create advisory committees pursuant to the Federal Advisory Committee Act (‘FACA’).^56^ The Vaccines and Related Biological Products Advisory Committee (‘VRBPAC’) is one such advisory committee for the FDA.^57^ Pursuant to the 2001 Federal Advisory Committee Act (FACA) Final Rule of 2001 at section 102–3.150, advisory committee meetings must be published in the Federal Register at least 15 calendar days prior to the meeting:

**§ 102–3.150 How are advisory committee meetings announced to the public?** 

(a) A notice in the **Federal Register** must be published at least 15 calendar days prior to an advisory committee meeting, which includes:
  (1) The name of the advisory committee (or subcommittee, if applicable);
  (2) The time, date, place, and purpose of the meeting;
  (3) A summary of the agenda, and/or topics to be discussed;
  (4) A statement whether all or part of the meeting is open to the public or closed; if the meeting is closed state the reasons why, citing the specific exemption(s) of the Government in the Sunshine Act, 5 U.S.C. 552b(c), as the basis for closure; and
  (5) The name and telephone number of the Designated Federal Officer (DFO) or other responsible agency official who may be contacted for additional information concerning the meeting.
(b) In exceptional circumstances, the agency or an independent Presidential advisory committee may give less than 15 calendar days notice, provided that the reasons for doing so are included in the advisory committee meeting notice published in the **Federal Register**.^58^

Pursuant to the statutory scheme, VRBPAC announced the Notice of a Meeting and established a public docket for the public to comment on EUA of the COVID-19 vaccine for children ages 5–11.^59^ The Notice was published on October 12, 2021, the comment due date was October 25, 2021, and the meeting was held on October 26, 2021.^60^ The October 26, 2021 meeting of the VRBPAC was available on youtube.com and is archived on youtube.com.^61^ The meeting lasted approximately 8 hours.

Toward the end of the 8-hour VRBPAC meeting, the committee voted on the following question: ‘Based on the totality of the scientific evidence available, do the benefits of the Pfizer-BioNTech COVID-19 Vaccine when administered as a 2-dose series (10 μg each dose, 3 weeks apart) outweigh its risks for use in children 5-11 years of age?’^62^ The VRBPAC voted in favor of the question.^63^

Upon recommendation of VRBPAC, the FDA authorized the Pfizer-BioNTech COVID-19 vaccine for children ages 5–11.^64^ The authorization meant that children were able to receive the vaccine. The FDA did not issue any sort of mandate, for example, nor does the FDA have the authority to mandate a vaccine in children.^65^ The role and authority of the FDA is likely misunderstood by some members of the public, who, quite understandably, may not be aware of the intricate regulatory scheme governing agencies and advisory committees.

### III.B. The Role of Public Comments

Public comments are a requirement of the APA’s rulemaking procedure.^66^ As described in the legal literature, the purposes of public comments include: (i) allowing the agency to obtain information from private parties who may have expertise in the regulated area and (ii) allowing for participation by the public, which is particularly important given that the agency decision is carried out by non-elected officials.^67^ Within rulemaking, the effectiveness of the notice and comment process is debated.^68^

The VRBPAC is an advisory committee and thus the debate about notice and comment as it pertains to agency rulemaking is different; however, it is still instructive given that VRBPAC initiated a public participation process allowing comments prior to its meeting. For the same reasons that some value can be found in APA 553’s requirement of notice and comment for agency rulemaking, the value of public participation can be applied to the VRBPAC meeting as well.^69^ The ability to comment prior to the convened meeting of VRBPAC allowed for the agency to obtain information from private parties who may have expertise and it allows for participation by the public.

Reverting back to the APA section 553 notice and comment scheme for the moment, it is noted that, sometimes, a proposed agency rule will be highly technical in nature and/or will generate very few comments.^70^ In this scenario, it can be argued that the public comment component does not serve a useful purpose because the party or parties who would comment are the same party or parties that are in close contact with the agency already.^71^ Thus, the agency is not obtaining new information and the public does not show a desire for participation. But, if the comment scheme could generate many comments, then this weighs in favor of the public participation reason to open a docket for public comments.^72^

The VRBPAC notice of meeting generated over 130,000 comments. This suggests, at the very least, that the public sought to participate in the COVID-19 vaccine authorization process. Thus, the normative reason to allow public participation in agency action, even if here an advisory committee meeting, is at the very least supported. Given the number of comments, it seems that studying the underlying reasons in the comments may provide value and insight.

Accepting for the moment that the generation of over 130,000 comments demonstrates support for the normative goal of public participation, this begs the question as to whether the VRBPAC obtained information from private parties who had expertise in this area that could help inform the committee decision. This is a more complicated question. Although discussed in more detail below, the present study analyzed a subset of the comments and found that within the subset, the overwhelming majority opposed EUA of the COVID-19 vaccine for children ages 5–11, but the VRBPAC voted in support of recommending EUA of the COVID-19 vaccine. This raises a question as to whether the VRBPAC obtained (or needed to obtain) expertise from private individuals with expertise in the regulated area. More narrowly, perhaps it can be argued that this is a highly technical area, and the committee did not need new information.

Regardless of whether the comments provided needed outside expertise, the sheer number of comments demonstrates that members of the public sought to be involved and have their voices heard, which is a hallmark of a democratic society. Given the large number of comments, this provides an opportunity to study public comments and attempt to understand the reasons or motivations behind such comments. The present study, described in detail in Section IV below, was intended to understand whether commenters were in favor of, neutral, or against EUA of the COVID-19 vaccine for children. Within those comments, we analyzed whether we could discern the underlying reason for the comment drawing on risk perception research.

## IV. EMPIRICAL STUDY

### IV.A. Methods

#### i. Comments analyzed

On October 12, 2021, the FDA issued a request for comments on Docket FDA-2021-N-1088 titled ‘Vaccines and Related Biological Products Advisory Committee; Notice of Meeting; Establishment of a Public Docket; Request for Comments’ (https://www.regulations.gov/document/FDA-2021-N-1088-0001). The FDA received 143,501 comments, although the public docket on Regulations.gov indicates that the related comments total ‘130.47 K’,^73^ and 1500 random numbers from 1 to 130,000 were generated, and the comments corresponding to those numbers based on order of submission were pulled using an API key received from the federal government.^74^ In consultation with the FDA, the API key was used in lieu of a Freedom of Information Act (‘FOIA’) request.^75^ The text of the comments was then transferred to a spreadsheet; any attachments to those comments were not pulled. Of the 1500 comments, two were not analyzed because there was no text, just an attachment. Fifty of the remaining 1498 comments were utilized by the coding team for a pilot coding exercise, leaving 1448 comments for coding and data analysis.

#### ii. Categories

Five categories were coded. The first category was coded to determine whether the commenter provided their name or initials (coded as 1) or whether the commenter was anonymous (coded as 0). The second category included emotional/affective words, such as mandate, death, killed, danger, coerced, forced, obligate, or require.^76^ If such a word was in the comment (or derivation of the word in past tense, for example), the comment was coded as 0. If no affective word was included, the comment was coded as 1. The third category was the general sentiment regarding whether the vaccine should be authorized. If the commenter was opposed to authorization, the comment was coded as 0. If the comment was neutral, such as simply requesting additional information, the comment was coded as 1. If the comment supported authorization, the comment was coded as 2. The fourth category included an ambiguity risk assessment as to whether the commenter indicated that the vaccine is more dangerous than the COVID-19 disease.^77^ If the commenter expressed a sentiment that the vaccine is more dangerous than the disease, the comment was coded as 0. If the comment did not reflect this sentiment, the comment was coded as 1. The fifth category included another ambiguity risk assessment as to whether missing information, such as the experimental nature of the vaccine or that the vaccine has not been studied long enough was expressed in the comment. If the commenter expressed a sentiment that the vaccine was experimental or not enough was known, then the comment was coded as 0. If the commenter did not express the sentiment, the comment was coded as 1. Table 1 summarizes the coding categories.

**Table 1:** Summary of coding categories

Category	Coding		
1. Anonymity	0 = anonymous	1 = name or initials	
2. Affect	0 = affective word used	1 = no affective word used	
3. Position re EUA	0 = oppose EUA	1 = neutral re EUA	2 = support EUA
4. Ambiguity (conflicting)	0 = risk re vaccine/disease	1 = no risk sentiment expressed	
5. Ambiguity (missing)	0 = risk re experimental	1 = no risk sentiment expressed	

#### iii. Interrater reliability and resolution of discrepancies

Four research assistants coded the 1448 comments. At least two research assistants coded each comment. Categories 2–5 required some judgment on the part of the coder; thus, an interrater reliability assessment was performed.^78^ Interrater agreement was 88.12 per cent for category 2; 93.99 per cent for category 3, 87.36 per cent for category 4, and 83.36 per cent for category 5. After the research assistants coded the comments and interrater reliability was evaluated, any discrepancies in coding were resolved by the first author (JKS) prior to subsequent analyses.

### IV.B. Data Analysis

All analyses were conducted using Stata 15.0 (StataCorp LLP, College Station, TX).

### IV.C. Results

Of the 1448 coded comments, only five of these comments were in favor of FDA authorization of the COVID-19 vaccine for children ages 5–11. Twenty comments were coded as neutral, and 1423 comments were coded as opposed to FDA authorization. Due to the small number of in favor or neutral comments, statistically significant comparisons among these categories could not be assessed. However, the frequency of coding categories provided an opportunity to analyze any trends.

We analyzed the extent to which pro/neutral/anti comments fell into other coding categories. We found that all types of comments (pro/neutral/anti) used words that indicate an underlying affective term. Results of the frequency are shown in Table 2.

**Table 2:** Frequency of overlap of category 3 with category 2

Category 3 (Position re EUA)	Total responses in Category 3 (Position re EUA)	% also coded 0 in category 2 (Affect)
Oppose EUA	1423	44.06%
Neutral re EUA	20	75%
Support EUA	5	40%

The results are interesting in that affective words were found in all category 3 comments. While the neutral category had the highest percentage of affective word use, this category was quite small, with only 20 comments. The commenters in support of EUA and those that opposed EUA used affective words in a similar frequency. However, only 5 commenters supported EUA, so this percentage is based on a very small number. What is apparent by the results is that ~44 per cent of the commenters who opposed EUA also used affective words. Given the large number of commenters that opposed EUA, this provides some insight that the ‘faint whisper of emotion’ may underlie some of the reasons for opposition to EUA. In other words, a misperception of risk may exist.

An example of a comment that opposed EUA and used affective words include (reproduced with syntax errors that are in the database):

Comment 32695: “Dear FDA (To Whom It May Concern)<br/>I am writing to ask you, please do not approve the covid-19 vaccines (or any vaccine) for children or for anybody else until the vaccines have been properly tested and with sufficient time to ensure safety. Anthony Fauci himself was recorded on a news interview in 2020 saying it would take at least a decade of trials and testing to ensure a new vaccine is safe.<br/>My family and I are against the 5-11 year old EUA as we are against any mandate for the covid-19 vaccines as there is insufficient safety data due to: their potentially hazardous rapid creation; inadequate clinical trials (*gave the control group the vaccine); and the super fast deployment process. <br/>Given more time, perhaps the initial studies could have found the tendency for Myocarditis in young males. According to research by the Covid 19 Early Treatment Fund, all three available vaccines in the U.S. have killed more people than they have saved in all age groups. (https://www.skirsch.com/covid/VCage.pdf). <br/>The EUA &ldquo;vaccines&rdquo; for COVID do not prevent contraction or spread of the virus. There are many unknowns such as how they will perform against future variants or other Sars type viruses. <br/>*In a February 2021 article by NPR, Dr. Steven Goodman, a clinical trials specialist at Stanford University, says losing those control groups makes it more difficult to answer some important questions about COVID-19 vaccines.<br/>&quot;We don't know how long protections lasts,&quot; he says. &quot;We don't know efficacy against variants &mdash; for which we definitely need a good control arm &mdash; and we also don't know if there are any differences in any of these parameters by age or race or infirmity.&quot; https://www.npr.org/sections/health-shots/2021/02/19/969143015/long-term-studies-of-covid-19-vaccines-hurt-by-placebo-recipients-getting-immuni<br/><br/>Please help cease any mandates with regard to the Covid 19 vaccines and please do not approve the vaccine for children or the mandates for children. <br/>Thank you for allowing me to express my views for your consideration.<br/>”^79^

An example of a comment that was neutral toward EUA and used affective words include:

Comment 6198: “As a Board Certified Child Psychiatrist I am gravely concerned about vaccinating children between the ages of 5-11 with the Pfizer vaccine. The grave concern arises from the known side effects from the vaccine. This vaccine is still experimental and the potential risks far outweigh the benefits. Specifically myocarditis in young males is very serious and this vaccine needs much more time to study before it is mandated to children. I am pro vaccine as long as it is tested adequately for safety.”

An example of a comment that supported EUA and used affective words include:

Comment 59886: “support a vaccine mandate for children if and when a vaccine for COVID-19 is approved for children.”

All three of these comments used the word ‘mandate’ (or a derivative thereof); however, the sentiment underlying the use of the word mandate varies. This shows a limitation of the methodology, in which the presence of affect was accounted for, but the valence of the affect was not. This observation will be discussed in Section V, below.

We also analyzed the frequency of the category of comments that fell into the category where the comments stated that COVID-19 is not a risk for children. Results of the frequency are shown in Table 3.

**Table 3:** Frequency of overlap of category of response with perception of risk of COVID-19 for children

Category 3 (Position re EUA)	Total responses in Category 3 (Position re EUA)	% also coded 0 in category 4 (Ambiguity – conflicting)
Oppose EUA	1423	39.92%
Neutral re EUA	20	15%
Support EUA	5	0%

The results are interesting in that the data indicate that commenters who opposed FDA authorization may have done so because they misperceive the risk of COVID-19 for children. Put differently, the commenters who opposed EUA may perceive that COVID-19 is not a risk for children or that the vaccine is riskier than the disease. None of the commenters who supported EUA also commented that COVID-19 is not a risk for children. A small percentage, 15 per cent, of 20 commenters who were neutral about EUA also included a sentiment that COVID-19 is not a risk for children. At the time that the FDA considered EUA for the COVID-19 vaccine for children ages 5–11, the risks to children of COVID-19 were less clear than they are today. Even though data indicated that children were at lower risk than adults for acute outcomes such as hospitalization and death, the risks for these outcomes are higher among unvaccinated children, and evidence suggests that vaccination of children is quite safe.^80^ Additionally, the risks of long-COVID and other biological changes remains unstudied.^81^ In other words, the science did not indicate that COVID-19 was not a risk for children because the science had not been done.

Examples of comments that opposed EUA and indicated a misperception of risk as to the risk of COVID-19 in children include (syntax errors in the database and reproduced below):

Comment 11145: “Please accept my opposition to mandatory vaccination of children, any age, against COVID 19. It is illogical to vaccinate children with shots that are 90% effective against a disease that the children have 99.8% natural immunity against. Thank you for your attention.”

Comment 118526: “Children should not be given an experimental vaccine with known side effects such as blood clotting and heart issues. It could also affect their future fertility. Children have a 99.9% recovery rate. Please use common sense.”

Comment 9185: “To put a child at risk, with no potential benefit is child abuse. These facts are well documented. There is not an excuse for ignorance. Children are more likely to die from the vaccine than covid19. This can not be for the &ldquo;greater good&rdquo;. That myth has been destroyed. You know this. We know this. Leave our children alone. You have destroyed enough lives. We are not lab rats.”

Comment 28792: “The risk is not worth the &quot;cure&quot; for my kids. The virus is almost no detected in kids by their symptoms. Why would we give them a vaccine with MANY side effects for something they are barely bothered or affected by. The potential risk for long term side effects in kids does not make sense to waste time worrying about. If mandated we will pull our kids from public schools and programs. If this is about health and safety, then vaccinating kids doesn't align.”

A limitation of the study is that unknown risks involve both unknown long-term risks of the vaccine and unknown long-term risks of COVID-19 infection; thus, unknown risks exist, and a commenter may be more concerned with one unknown risk over another. The context of the comment becomes important, including that ‘children are more likely to die from the vaccine than covid19’ is an example where the perception of risk did not align with the evidence-based risk assessment. This observation will be discussed in Section V, below.

Studies aimed at understanding the long-term effects of COVID-19 in children are underway.^82^ In addition, as stated by Purvi Parikh: ‘But there’s not a single infectious disease, actually, that has reached that status [referring to herd immunity] without a vaccine through the history of time[.]’^83^ Thus, the risk of COVID-19 in children is at best understudied. In addition, getting COVID-19 under control in the population requires vaccination.

We analyzed the frequency of the category of comments that also fell into the category that the experimental nature of the COVID-19 was among the reasons stated in their comment. Results of the frequency are shown in Table 4.

**Table 4:** Frequency of overlap of category of response with experimental nature of vaccine

Category 3 (Position re EUA)	Total responses in Category 3 (Position re EUA)	% also coded 0 in category 5 (Ambiguity—Missing)
Oppose EUA	1423	61.14%
Neutral re EUA	20	85%
Support EUA	5	100%

These results are interesting because the largest frequencies in overlap are found in this category. The underlying type of comment that referred to the experimental nature of the vaccine was quite different for those that supported EUA and those that opposed. For example, in support of EUA, the following examples of comments were made (syntax errors in database and reproduced below):

Comment 67392: “Please shrug off the nonsense so many are shouting at you about conspiracy theories they read on the Internet.<br/><br/>If the data indicates safety and efficacy, give us the tools we need to keep our children safe.”

Comment 81438: “If the COVID vaccine is deemed safe for 5-12 year olds, please make it mandatory for all children in this category to help keep them, and the rest of the population, safe.”

For those that opposed EUA, the following examples of comments were made (syntax errors in database and reproduced below):

Comment 102403: “Not only is it unnecessary to vaccinate these young children, but this would be a crime against humanity. The efficacy data does NOT prove these vaccines safe or effective for young kids. Protect the children!”

Comment 110620: “Covid vaccine for children 5-11 years old is not warranted. The science and data shows this is extremely dangerous to these individuals. There have not been enough trials to understand the long term effects on children. The risks of these vaccines significantly outweighs any benefits. Please reject the application to vaccine our children.<br/>” Comment 19867: “There are no adequate long-term safety studies of mRNA covid vaccines and the transmission of COVID-19 among children in schools and daycares is very rare.” Comment 31727: “I am writing regarding allowing Covid-19 shots for children between the age of 5-11. The American people are counting on you to apply the same rigorous standards you have applied to other immunizations. Since there have been no long term studies on these shots it is wrong to inject them into children who are at such low risk from COVID-19. There has been no fertility testing on these novel shots. Children should not be made into test subjects. This shot is particularly concerning for young men who are having cardiac side effects. If you apply the same standards that you always have before approving a Vaccine then I have no doubt you will do the right thing and not approve it for children between 5-11. They are counting on you to apply a sound scientific standard to this issue which may have extreme consequences in their lives, consequences that may only be clear later. In view of the very low risk that COVID-19 poses to them, and in view of the severe side effects that some are experiencing, and in view of the lack of fertility and long term testing, please do not approve them. <br/>Sincerely,<br/>[name redacted]”

At the time that the COVID-19 vaccine received EUA for children, hundreds of millions of doses of the vaccine had been given worldwide. Thus, the risk of the vaccine in a real-world setting was understood quite well. To some extent, the novelty of the use of mRNA might explain some of the aversion toward the vaccine.^84^ Admittedly, risk assessment is imperfect, but between understanding how vaccines work and the very low risk of the vaccine that had been given to millions of people, the risk assessment was quite attainable.^85^ This observation will be discussed in Section V, below.

We also analyzed how many of the comments that opposed EUA overlapped in two or more risk perception categories, and 830 of the comments, or 58.33 per cent, overlapped in two or more categories. In addition, 149 of the comments that opposed EUA, or 10.47 per cent, did not fall into any of the risk perception categories. In other words, most comments that opposed EUA fell into one of the risk perception categories.

## V. DISCUSSION OF LEGAL IMPLICATIONS

The results of our study demonstrate that most commenters who opposed EUA provided reasons that suggested misperception of risk, in general and/or relative to infection. Many comments in opposition to EUA utilized affective words or included explanations that suggested missing or conflicting information as part of their reasoning. Not surprisingly, many of the comments opposed to EUA combined affective and ambiguity aversion reasoning, indicating that decision-making is influenced by multiple simultaneous processes.

Understanding the underlying reasons for vaccine hesitancy is important to attempt to align individual assessment of risk with evidence-based assessment of risk. Legal interventions can be used to attempt to close this divide, but these interventions likely need to target perception of risk to obtain the desired result.^86^ This article discusses the need for interventions prior to policy implementation. The rationale for implementing an intervention prior to enactment of a policy is that the legal policy is more likely to be acceptable if individual perception of risk is in line with evidence-based risk assessment. Discussed below are two avenues for legal implications: (i) addressing risk perception prior to legal policy implementation and (ii) querying whether the notice and comment process could be a point of intervention to help align individual perception of risk with evidence-based assessment of risk. This section concludes with limitations of the study and possible avenues for future inquiry.

### V.A. Addressing Risk Perception prior to Legal Policy Implementation

Many types of legal interventions involve a risk/benefit or cost/benefit analysis prior to implementation.^87^ However, in society, we are seeing a rejection of these policies. In the COVID-19 pandemic, we saw/see rejection of mask mandates and vaccine mandates.^88^ In order for these policies to obtain the desired result, one approach is to consider individual’s decision-making processes as part of the legal intervention. For example, the term ‘mandate’ can elicit an affective response.^89^ Thus, ‘mask mandate’ or ‘vaccine mandate’ is less likely to obtain the desired result. Similarly, ‘forced’ can elicit an affective response; if individuals believe they will be ‘forced’ to take the vaccine, they may experience fear or dread and misperceive the risk of the vaccine.^90^ If an individual associates a vaccine with death or long-term disability, the individual may experience an affective response against the vaccine.

Interestingly, although the number of relevant comments was quite small, there was a hint that the term ‘mandate’ in this study could suggest a positive affective response as well. That is, mandating a vaccine during a pandemic could elicit positive feelings for those individuals who supported EUA of the COVID-19 vaccine. In this way, fear of the disease, fear of spread of the disease, or fear that others might not engage in efforts to protect themselves might elicit an affective response such that the individual’s assessment of risk is in line with evidence-based risk assessment of the COVID-19 vaccine. Similarly, the term ‘death’ or ‘die’ could, reasonably, be associated with the COVID-19 disease. Thus, commenters could use the word ‘death’ *vis à vis* the disease, and not the vaccine. This suggests that looking for particular words alone is insufficient; rather, the context, or valence, in which the affective words are used is needed to understand how an individual perceives risk.

Understanding how a particular word or message lands on an individual could form the basis of a possible intervention. For example, an affective word such as ‘death’ or ‘die’ likely elicits a negative affect, such as fear or dread. Connecting the word ‘death’ with the disease COVID-19 has a different connotation that connecting the word ‘death’ with the COVID-19 vaccine. Attempting to influence the quick-thinking about fear of the disease versus fear of the vaccine requires further investigation. Likewise, providing missing information or resolving conflicting information is a potential intervention strategy. If, for example, an individual hears that people ‘die’ from a vaccine, then this creates conflicting information with the evidence-based risk assessment. The conflicting information creates ambiguity, and the individual may assign a high risk to the vaccine. Learning that ambiguity exists and may be a possible reason for misperception of risk (as the current study suggests), allows for the development of possible interventions.

Put differently, an intervention can focus on the affective response or resolving ambiguity prior to legal policy implementation. Most of the comments evaluated in this study contained affective words or evidence of ambiguity aversion. Perhaps quick-thinking skills do not serve individuals well in this circumstance. As Paul Slovic noted: ‘The law must learn to tell the difference.’^91^ Possible interventions can focus on moving an individual to have a quick-thinking response that aligns with evidence-based risk perception.

Addressing risk perception related to vaccines is important in other areas beyond going so far as vaccine mandates. That is, even in the absence of a mandate, public health messaging around vaccination can utilize messaging that incorporates what we know about decision-making, including that people come to decision-making with biases.^92^ Risk communication is important to allow individuals to appropriately assign risk and should be differentiated from risk regulation.^93^ Put differently, multiple modes of risk communication strategies, which can occur before, during, or after legal policy implementation are important. The present study analyzed public comments, thus the discussion herein is focused on this narrow avenue for possible intervention.

### V.B. Notice and Comment Process as a Point of Intervention

Another possible utilization of this study is to evaluate the utility of public comments. As discussed earlier, one reason for public comments is to allow the public to engage with agency decision-making as part of democratic values.^94^ In the study described herein, certainly members of the public sought to engage. The notice for public comments elicited an enormous response—with over 130,000 comments. This is atypical of the number of comments normally received on a request for public comments.^95^ It seems that if the public seeks to engage, then they should be allowed to do so as part of our democratic values. The observation begs the question as to whether public comments serve the purpose they were intended to serve.

The role and value of public comments is debated. Academic commentary on the role of public comments shines different lights. As noted by E. Donald Elliott:

The primary function of the notice-and-comment rulemaking process in our system has shifted since the enactment of the Administrative Procedure Act (APA) in 1946. What was once (perhaps) a means for securing public input into agency decisions has become today primarily a method for compiling a record for judicial review. No administrator in Washington turns to full-scale notice-and-comment rulemaking when she is genuinely interested in obtaining input from interested parties. Notice-and-comment rulemaking is to public participation as Japanese Kabuki theater is to human passions – a highly stylized process for displaying in a formal way the essence of something which in real life takes place in other venues. To secure the genuine reality, rather than a formal show, or public participation, a variety of techniques is available-from informal meetings with trade associations and other constituency groups, to roundtables, to floating “trial balloons” in speeches or leaks to the trade press, to the more formal techniques of advisory committees and negotiated rulemaking. The notice-and-comment process is not worthless. It fulfills an important function-to compile a record for judicial review-not primarily to provide public input into government thinking, contrary to the assumption of many academics.^96^

While an academic debate regarding the utility of the notice and comment process centers, in part, on whether an agency followed the correct procedural avenue for choosing to use guidance versus the informal rulemaking process, for example, the underlying discussion regarding the utility of public comments may be applicable to other scenarios, such as the case herein.^97^ Here, the question is whether the notice and comment process for the advisory committee served a useful purpose—either for the advisory committee or for the public.

As observed by Edward Rubin, the core of the APA relies on participation by private parties, either through public comments, private communications with the agency, or through private parties initiating legal challenges.^98^ Participation by private parties typically involves large organizations, including labor unions, large interest groups, and businesses.^99^ As observed by Rubin, ‘[v]very few people other than professional lobbyists or lawyers employed by such organizations read the Federal Register, send comments to agencies regarding proposed rules, or challenge the legality of such rules in federal court.’^100^ This calls the role of individuals in the notice and comment process into question or perhaps raises questions about the value of individual participation.

In light of Professor Rubin’s observations about the participation of private parties in the comments process, one may question whether there might have been a ‘call to action’ or some sort of movement to tell members of the public to comment on this particular notice of meeting.^101^ It is possible that an anti-vaccine organization or an anti-vaccine advocate informed members of the public about this particular notice for comments and suggested that their followers engage. This is pure speculation, and it begs the question as to whether an investigation into whether the anti-vaccine movement is spurring on such a response.

Another observation about this study is that the overwhelming number of comments opposed EUA (as based on our sample size); however, the advisory committee supported EUA. It did not appear that the public comments provided information to the advisory committee that they did not already know. Vaccine efficacy and safety is a highly technical area, and thus, it is reasonable to speculate that the public comments could not provide information that the advisory committee did not already know.^102^ But, if the public seeks to participate, it seems that it should provide some role. Could the role or value be that it serves a reverse purpose—that is, it is a way for the agency to educate the public? This is not a traditional purpose of the notice and comment process, but perhaps it could become a possible role for the notice and comment process, at least in controversial areas.

In this case, the published notice for the meeting was a very short document.^103^ Nothing in the notice provided any scientific information or any educational information. The notice itself may have met regulatory requirements, but it is possible that some form of education could be part of the Notice. In any event, the regulations do not appear to prohibit the release of educational material. One possible way to educate the public is to include information in the Notice of Meeting that provides valuable information. Secondarily, utilizing risk communication strategies might also provide an approach as to how the information is presented.

Another observation from the comments analyzed suggests that members of the public may not fully understand the role of the FDA.^104^ The FDA does not mandate vaccines, for example, such that the comments supporting or opposing the EUA that focused on mandating for forcing a vaccine were misplaced, at least from the regulatory aspect.^105^ It is true that once the vaccine receives EUA, that state and federal governments can require vaccination, for example. But that is a separate process controlled by different agencies. Again, this suggests a possible reason to open a dialogue to educate the public about the role of agency action.

An important observation of this study is that members of the public seeking to participate in a regulatory process are doing so based on a misunderstanding of the issues being regulated. That is, regardless of agency action and subsequent policy implementation, members of the public misperceive the risk of the COVID-19 vaccine, especially relative to the risk of the disease. The results of this study suggest that affect and ambiguity may contribute to misperception of risk. It is more than just a misunderstanding of facts or believing misinformation; it is a behavioral decision-making process.

The public comments in this case are instructive in thinking about regulating risk perception prior to enactment of any legal policy. One question is whether the public comment process could be utilized such that regulators (or their advisors) can engage with the public to close the divide between individual misperception of risk and evidence-based assessment of risk.^106^ As suggested above, one strategy is to include meaningful information in the Federal Register. Another suggestion is to hold a pre-meeting to the meeting in which information is presented in a way that is more likely to allow individuals to appropriately assign risk. A third strategy could be to require people who publicly comment to move through a questionnaire or other information sharing procedure prior to posting their comment.^107^ That does not appear to be the way that public comments are utilized as of now, but that does not mean that it cannot be so in the future. Importantly, the public has an understandable right to participate. The question is how to make that participation the more valuable for all parties. Admittedly, a limitation of the above suggestions is that they create barriers to participation because they might involve more time on behalf of the individual, but working through these suggestions might highlight ways to marry education with public participation.

The number of comments on this notice of meeting suggests that the public wants to participate. It follows that this provides an opening to engage and educate members of the public on risk regulation. Given that most comments were associated with affective words and/or statements that suggest ambiguity aversion, this suggests that interventions that utilize these decision-making theories could be considered.^108^

For example, is there a way to engage with individuals who comment to resolve missing or conflicting information? If an individual comments in a way that suggests they received information that more people die from the vaccine as compared to the disease, then this creates conflicting information when the FDA (or its advisory committee) provides information that the vaccine is safe and effective. Ambiguity aversion informs us that when individuals receive conflicting information, they are more likely to assign a high risk and low benefit. It seems the right to comment and engage is not enough for a particular individual and thus the commenting process does not (nor is it intended to) function as an intervention. Perhaps an intervention that resolves missing or conflicting information as part of the lead-up to the right to comment could have an impact. Future studies could attempt to test this idea.^109^

Is there a way to engage with individuals who comment to resolve their feelings of fear or dread associated with the COVID-19 vaccine? Many commenters in our study utilized words that suggest an affective response to the COVID-19 vaccine EUA that suggests fear or dread of the vaccine. In contrast, a small minority of commenters appeared to view the EUA as a welcome addition to alleviating the pandemic (suggesting positive or good emotions). At present, the comment process is not designed to resolve discrepancies between commenters, or between perceived and empirical risk. Is it plausible that it could be re-designed to have this impact?

We do not know whether the approximately 8-hour meeting of the Advisory Committee alleviated the concerns of those that opposed EUA, nor even how many commenters watched the meeting.^110^ It is possible that the meeting served as a form of intervention and did resolve the perceived risk of the COVID-19 vaccine at least for some commenters. It is also possible that it did not.

The discussion of possible changes to the notice and comment process requires additional study in an experimental setting to determine whether the possible changes would have a desired effect. A limitation of a public education campaign prior to posting a comment could be that it decreases public participation, although this would need to be evaluated through a pilot study, for example.

Another limitation is understanding how many people know about the comment process, let alone participate in it. Dramatic changes to the notice and comment process might only reach a small segment of the population, although, again, pilot studies would need to analyze that anticipated problem. Given academic literature criticizing the notice and comment process, broadly speaking, changes in some form might bear fruit to address multiple concerns, including the ones raised in this article.

For example, qualitative focus group studies could be conducted to obtain further understanding about how notice and comment information is interpreted by the general public and how to better mitigate concerns. Utilizing these findings, pilot studies might be designed to manipulate different factors, such as the availability of educational material that resolves missing or conflicting information, or the affective impact of the information presented. Statistical analyses could then evaluate specific hypotheses; e.g. whether participants’ likelihood of supporting specific FDA rules might differ depending on the extent to which key information is either missing or ambiguous, or the participants’ affect, or other factors of interest. Ultimately, this line of research could lead to the refinement of the notice and comment process to incorporate interventions that are tailored to the individual respondent and designed to reduce the likelihood that participants misperceive risk.

Overall, the pilot studies described above allow for the testing, both quantitatively and qualitatively, as to whether changes in how additional information is provided the notice and comment process may impact how people comment. These focus group studies could also have questionnaires asking participants whether going through additional steps of receiving information poses a barrier to them in the comment process. In sum, changes to the notice and comment process with the goal to make the process more meaningful to the participants, can be tested through studies that aim to determine whether additional information assists in decision-making and whether the additional steps are helpful or create potential barriers to participation. These studies can assess, at least preliminarily, the trade-offs and limitations as discussed herein.

Given the low rates of vaccination of children ages 5–11, this suggests that many parents are hesitant to vaccinate their children. Perhaps these parents do not fear the COVID-19 disease for their children or perhaps these parents do not fear community spread.^111^ We know from some of the comments that commenters did not think that children would get the disease at school or day care, though seroprevalence studies suggest that approximately 75% of children have had COVID-19 at least once.^112^ This suggests significant community spread, which implicates congregate settings such as schools and daycares.

We also do not know the long-term impact of COVID-19 on children.^113^ Interestingly, one of the reasons that commenters made in opposition to the EUA was uncertainty regarding long-term effects of the COVID-19 vaccine. It is notable that concerns about unknown long-term effects of the vaccine motivated some EUA opposition, suggesting an aversion to missing information, while similar uncertainly regarding the long-term effects of COVID-19 did not appear to weigh the same on commenters.^114^ Of note, the long-term effect, or lack thereof, could also be an affective response, that is fear of a serious long-term effect. Again, it is interesting to observe that commenters appeared to fear the unknown long-term effects of a vaccine and not the unknown long-term effects of COVID-19.

Scientists are much more familiar with vaccines and studying vaccines as compared to COVID-19 and studying COVID-19. The risk calculation for the long-term effect(s) of a vaccine is likely more ascertainable as compared to the disease.

For COVID-19, we know some of the potential areas of concern for long-term impacts. One study, for example, found that every single person who had COVID-19 experienced brain injury.^115^ Additional studies have analyzed changes to brain microstructure months and years following COVID-19 infection.^116^ In one study, reversible changes to brain structure were observed at a 2-year follow-up.^117^ Another study found that everyone who had COVID-19 had changes in metabolism and Body Mass Index (BMI).^118^ However, another study did not detect significant changes in BMI at a follow-up study.^119^ These types of studies suggest that significant changes occur to various organs because of COVID-19, although studies suggest that the body may be able to recover. To date, no study suggests such dramatic changes to organs because of the COVID-19 vaccines. This does not mean that vaccines pose no risk, there is some risk of myocarditis, for example.^120^ But, it does suggest that the risk of the vaccine is likely lower than the risk of disease, at least in the short term and possibly in the long term as well.^121^

This study supports the reality that the FDA can receive public comments and still base its decision on evidence-based risk assessment. But this might beg the question as to the utility of the public comments, at least in such a technical area as vaccine regulation and authorization. What is the value of public comments in such a technical area? Without truly educating the public first, it is possible that the comments do not have much value.^122^

### V.C. Limitations and Possible Future Directions

This study is not without limitations. For the affective word category, it is possible that not all endings of words were captured in the coding, for example, the ending ‘ibly’ may not have been included for all words in the category. Thus, this category might be underinclusive. In addition, the term die (or a variant thereof) was often used in other contexts not necessarily the vaccine for children, so this category can be overinclusive. Another major limitation is that the coding in some categories required judgment, which is why multiple coders coded each category. Discrepancies in coding were resolved by the first author. Even with good inter-reliability indicators, as was the case here, it is possible that some coding might not be the same under all circumstances. Another limitation is that there were so few comments supporting EUA that comparisons between the categories of comments for those that supported and opposed could not be made. While it is an interesting observation that most commenters (at least in our sample) opposed EUA, it did not allow for a comparison among groups. Finally, another limitation is that the commenters might be an unrepresentative sample because those that agreed with the expected outcome of the vaccine advisory meeting were unlikely to comment; perhaps only those that were upset about the expected outcome chose to comment.^123^

We also learned that the term ‘mandate,’ for example, may be context specific. For those that support the vaccine, the term mandate may elicit positive feelings, whereas those that oppose the vaccine, it may elicit negative feelings. The same observation can be made with the words ‘danger’ or ‘death’ in that the context in which the word is used matters. In other words, the coding alone does not provide the full story for each affective word in each comment; the presence of affect was accounted for, but the valence of the affect was not.

In the ambiguity aversion categories, the comments do not necessarily have a black-and-white demonstration of a preference for known risks over unknown risks. Scientists have long understood the mechanism of acquired immunity via vaccines and have ability to assess risk, although admittedly no risk assessment is perfect and holds for decades to come. On the other hand, the long-term risks of COVID-19 for children are unknown because scientists do not have the long-term data to understand the mechanism and consequences of infection. Nonetheless, a limitation of the study is that unknown risks exist. The analysis of the comments suggests avenues for continued research on ambiguity aversion and vaccine hesitation.

Another interesting observation, although not necessarily surprising, was the overlap between the affective category and the ambiguity aversion categories. Even if it is not possible to discern between those who support EUA and those who oppose, it is an interesting observation that affect and ambiguity appear to play a role in risk perception of EUA of the COVID-19 vaccine for children.

A final limitation is that this is an observational study on a database that was created outside of an experimental setting. As a result, the ability to use predictors to determine whether a commenter had a general aversion to ambiguous information could not be used to interpret their comment and was not available for statistical analysis.^124^ But, the study contained herein could be used as a launching pad for future studies in an experimental setting. One possibility is to contact commenters who provided their names and request participation in a future study. Another possibility is to tease out the context-dependent use of words or sentiments to control the context in an experimental setting.

One question for future investigation is whether the notice and comment process can be used as a point of intervention to educate individuals on a technical issue. Although it would not be feasible (nor should it be) in many areas to do an educational campaign, only a small number of notice and comment opportunities elicit large numbers of comments. When they are anticipated to do so or they do, this seems like an opportunity to use the process to attempt to align individual’s perception of risk with evidence-based assessment of risk. At least, when a notice in the Federal Register starts eliciting hundreds or even thousands of comments, this seems like a time for experts in the area to educate the public and conduct some pre-legal policy risk regulation.

Most of the comments in the current study were not anonymous (1049 comments were not anonymous; 399 were anonymous). Is it possible to conduct individual outreach to individuals who submit public comments in the few cases where a notice generates so many public comments? Or, at least, it could allow for contact to see if commenters are willing to engage in focus groups to assess any strategies geared toward appropriate risk assessment.

In sum, the present study provided interesting information that most comments that opposed EUA of the COVID-19 vaccine for children ages 5–11 were associated with a misperception of risk. These data provide insight as to why COVID-19 vaccine hesitancy, particularly in pediatric populations, exists. This article suggests possible points for intervention to close the divide between misperception of risk and evidence-based assessment of risk.

## VI. CONCLUSION

The present study analyzed public comments on a notice of meeting of the vaccine advisory committee for the FDA to determine whether risk perception theories might help explain why individuals opposed emergency use authorization for the COVID-19 vaccine for children ages 5–11. We found that most comments fell into one or more categories that suggest affect or ambiguity may form the basis of the position taken by the commenter. Understanding why individuals inappropriately assign risk as compared to evidence-based assessment of risk is important as we face major world-wide issues, such as the COVID-19 pandemic and other major challenges, such as climate change. This study, perhaps secondarily, allows for analysis about the value and role of public comments. If one purpose of public comments is to allow the public to engage in agency decision-making, then perhaps future studies should investigate how to make that interaction more productive and meaningful.

## Footnotes

1 See Kaiser Family Foundation, *KFF COVID-19 Vaccine Monitor*, https://www.kff.org/coronavirus-covid-19/dashboard/kff-covid-19-vaccine-monitor-dashboard/ (accessed July 31, 2023) (showing a diagram of these who said ‘they will get COVID-19 vaccine as soon as they can’ and a ‘wait and see’ group).

2 One definition of vaccine hesitancy is a ‘delay in acceptance or refusal of vaccination despite availability of vaccination services. Vaccine hesitancy is complex and context specific, varying across time, place and vaccines’. Noni E. MacDonald, SAGE Working Group on Vaccine Hesitancy, *Vaccine hesitancy: Definition, scope and determinants*, 14 Vaccine, 4161, 4161 (2015). Vaccine refusal is defined as ‘[u]willingness to allow oneself or a family member to be immunized against a preventable contagious disease, such as measles, mumps, rubella, or chickenpox. It occurs most often in people who fear adverse effects from vaccination, in people who have religious or philosophical objections to vaccination, and in people who have had allergies to a component of a vaccine’. Medical Dictionary, *vaccine refusal*, https://medical-dictionary.thefreedictionary.com/vaccine+refusal (accessed July 31, 2023).

3 Food and Drug Administration, *FDA Authorizes Pfizer-BioNTech COVID-19 Vaccine for Emergency Use in Children 5 through 11 years of Age*, https://www.fda.gov/news-events/press-announcements/fda-authorizes-pfizer-biontech-covid-19-vaccine-emergency-use-children-5-through-11-years-age (last updated Oct. 29, 2021) (‘The FDA has determined this Pfizer vaccine has met the criteria for emergency use authorization. Based on the totality of scientific evidence available, the known and potential benefits of the Pfizer-BioNTech COVID-19 vaccine in individuals down to 5 years of age outweigh the known and potential risks.’).

4 Lois A. Weithorn & Dorit Rubenstein Reiss, *Legal Approaches to Promoting Parental Compliance with Childhood Immunization Recommendations*, 14 Human  Vaccines & Immunotherapeutics 1610, 1610 (2018) (‘Parents who refuse vaccinations for their children are often influenced by misleading characterizations of vaccine risks promulgated on the internet and in the media.’).

5 See, e.g. Dan M. Kahan, Kathleen Hall Jamieson, Asheley Landrum & Kenneth Winneg, *Culturally Antagonistic Members and the Zika Virus: An Experimental Test*, 20 J. of  Risk  Research 1, 1–2 (2017) (describing the dynamics of the affect heuristic and cultural cognition).

6 Daniel Ellsberg, *Risk, Ambiguity, and the Savage Axioms*, 75 Q. J. Econ., 643, 657, 659 (1961) (‘Let us assume, for purposes of discussion, that an individual can always assign relative weights to alternative probability distributions reflecting the relative support given by his information, experience and intuition to these rival hypotheses. This implies that he can always assign relative likelihoods to the states of nature. But how does he *act* in the presence of his uncertainty? The answer to that may depend on another sort of judgment, about the reliability, credibility, or adequacy of his information (including his relevant experience, advice and intuition) as a whole: not about the relative support it may give to one hypothesis as opposed to another, but about its ability to lend support to any hypothesis at all’.).

7 Paul Slovic, *What’s Fear Got to Do with It? It’s Affect We Need to Worry About*, 60 Mo. L. Rev. 971, 977 (2004) (hereinafter ‘Slovic 2014’).

8 See Sheila Jasanoff, *Complete Separation of the Two Processes is a Misconception*, 19 EPA J. 35 (1993) (discussing the relationship between risk assessment and risk management).

9 See, e.g. Lozano Smith, *Superior Court Ends Lawsuit Challenging Face Mask Mandate in California Schools*, (Dec. 22, 2021), https://content.acsa.org/superior-court-ends-lawsuit-challenging-face-mask-mandate-in-california-schools/ (‘Of particular interest, the court found that the State’s face covering requirement in the K-12 school setting was valid under California law, as was the mandate’s obligation to exclude students that refused to comply with the mask requirement. Correspondingly, the exclusion of students from school for refusal to wear a face mask does not constitute a suspension or expulsion in violation of Education Code section 48900. Rather, the court found that there is a difference between exclusion of students for public health reasons and suspension or expulsion for disciplinary reasons’.).

10 See, e.g. Micah D.J. Peters, *Addressing Vaccine Hesitancy and Resistance for COVID-19 Vaccines*, 131 Int’l J of  Nursing  Studies 1, 4–6 (discussing vaccine hesitancy and vaccine refusal).

11 Food and Drug Administration, *Vaccines and Related Biological Products Advisory Committee; Notice of Meeting; Establishment of a Public Docket; Request for Comments*, FDA-2021-N-1088-0001, (posted Oct. 12, 2021), https://www.regulations.gov/document/FDA-2021-N-1088-0001 (hereinafter ‘COVID-19 vaccine docket’).

12
*Id*. In communications with the FDA, the first author learned that Regulations.gov did not have the technological capacity to post all the comments, although all the comments are publicly available through other methods. Private communication via telephone conversation.

13 Food and Drug Administration, *Emergency Use Authorization for Vaccines Explained*, (Nov. 20, 2020) https://www.fda.gov/vaccines-blood-biologics/vaccines/emergency-use-authorization-vaccines-explained.

14
*Id*. (‘In public health emergencies, such as a pandemic, the development process may be atypical. For example, as demonstrated by the response to the COVID-19 pandemic, the U.S. government has coalesced government agencies, international counterparts, academia, nonprofit organizations and pharmaceutical companies to develop a coordinated strategy for prioritizing and speeding development of the most promising vaccines. In addition, the federal government has made investments in the necessary manufacturing capacity at its own risk, giving companies confidence that they can invest aggressively in development and allowing faster distribution of an eventual vaccine. However, efforts to speed vaccine development to address the ongoing COVID-19 pandemic have not sacrificed scientific standards, integrity of the vaccine review process, or safety’.).

15 As described in Section IV, *infra*, the public docket for comments was for the vaccine advisory committee to the FDA, but the FDA adopted the recommendation from its vaccine advisory committee. It is possible that some commenters differentiated between the vaccine advisory committee and the EUA for the FDA, but such differentiation was not seen in the comments analyzed.

16 In other words, whether the affect heuristic helps us to understand why individuals might oppose EUA. See Slovic 2014, *supra* note 7, at 976–7 (2004) (discussing early research on dread and outrage in risk perception).

17 In other words, whether ambiguity aversion helps to explain why individuals might oppose EUA. See Ellsberg, *supra* note 6, at 657, 663, 666 (1961) (‘In contrast, the ambiguities surrounding the outcome of a proposed *innovation*, a departure from current strategy, may be much more noticeable’.).

18
Daniel  Kahneman, Thinking  Fast and  Slow 137–45 (2011) (‘Paul Slovic probably knows more about the peculiarities of human judgment of risk than any other individual. His work offers a picture of Mr and Ms Citizen that is far from flattering: guided by emotion rather than by reason, easily swayed by trivial details, and inadequately sensitive to differences between low and negligibly low probabilities’.).

19 Slovic 2014, *supra* note 7, at 971.

20
*Id*.

21
*Id*.; Kahneman, *supra* note 19, at 114 (‘*System 1* operated automatically and quickly, with little or no effort and no sense of voluntary control. *System 2* allocates attention to the effortful mental activities that demand it, including complex computations. The operations of System 2 are often associated with the subjective experience of agency, choice, and concentration’.).

22 Kahneman, *supra* note 19, at 58.

23 Slovic 2014, *supra* note 7, at 976; Paul Slovic, Melissa L. Finucane, Ellen Peters & Donald G. MacGregor, *The Affect Heuristic*, 177 Eur. J. of  Operational  Res. 1333, 1342 (2007) (‘This is the finding that judgments of risk and benefit are negatively correlated. For many hazards, the greater the perceived benefit, the lower the perceived risk and vice versa’.) (hereinafter ‘Slovic 2007’).

24 Slovic 2014, *supra* note 7, at 977 (‘The significance of this finding for the affect heuristic was not realized until a study by Alhakami and Slovic found that the inverse relationship between perceived risk and perceived benefit of an activity (e.g., using pesticide) was linked to the strength or positive or negative affect associated with that activity as measure by the rating of the activity on bipolar scales such as *good/bad, nice/awful, dreaded/not* dreaded, and so forth’.); Ali Siddiq Alhakami & Paul Slovic, *A Psychological Study o the Inverse Relationship Between Perceived Risk and Perceived Benefit*, 14 Risk  Analysis 1085, 1094–5 (1994) (addressing the inverse relationship between risk and benefit calculations and finding that ‘[a] person’s general affective evaluation of the item was the major predictor of the risk/benefit correlation’.).

25 Slovic 2014, *supra* note 7, at 977.

26 Slovic 2007, *supra* note 24, at 1344 (2007); Paul Slovic & Ellen Peters, *Risk Perception and Affect*, 15 Current  Directions in  Psychol. Sci. 322, 322–3 (2006) (hereinafter ‘Slovic & Peters 2006’); Edward Royzman, Corey Cusimano & Robert F. Leeman, *What lies beneath? Fear vs. Disgust as Affective Predictors of Absolutist Opposition to Genetically Modified Food and Other New Technologies*, 12 Judgment and  Decision  Making 466, 472–4 (2017); Michael Siegrist & Bernadette Sütterlin, *Human and Nature-Caused Hazards: The Affect Heuristic Causes Biased Decisions*, 34 Risk  Analysis 1482, 1489, 1491–2 (2014).

27 Paul Slovic, *Perception of Risk*, 236 Sci. 280, 285 (2017).

28 Slovic & Peters 2006, *supra* note 27, at 324.

29 Slovic 2014, *supra* note 7, at 983.

30
*Id*. The New York Times publishes the average new deaths from COVID-19 each day on website. On September 19, 2022, for example, the New York Times reported 461 new deaths from September 18, 2022. *Coronavirus in the U.S.: Latest Map and Case Count*, https://www.nytimes.com/interactive/2021/us/covid-cases.html (accessed Sept. 19, 2022) (on Sept 7, 2022, the New York Times reported 829 new deaths with the daily average of deaths at 397; on Sept. 18, the New York Times reported 8 new deaths and a daily average of 464 deaths. It is not completely clear how the New York Times reconciles these numbers.).

31 Slovic 2014, *supra* note 7, at 990.

32
*Id*.

33 Ellsberg, *supra* note 6, at 666 (‘The decision rule will not preclude such an act, but it will definitely bias the choice away from such ambiguous ventures and toward the strategy with the “*known* risks.”’).

34
*Id*. at 657.

35
*Id*. at 659–61, 666 (‘Thus the rule is “conservative” in a sense more familiar to everyday conversation than to statistical decision theory; it may often favor traditional or current strategies, even perhaps at high risk, over innovations who consequences are undeniably ambiguous’.).

36
*Id*. at 662–664.

37
*Id*. at 666–7.

38 Elisa Cavatorta & David Schröeder, *Measuring Ambiguity Preferences: A new Ambiguity Preference Survey Module* 58 J. of  Risk and  Uncertainty 71, 72 (2019).

39 Laura L. Blaisdell, Caitlin Gutheil, Norbert A. M. Hootsman & Paul K. Han, *Unknown Risks: Parental Hesitation about Vaccination*, Med. Decision  Making 479, 479 (2016).

40
*Id*. 479–80, 483 (one response from a member of the focus group stated: ‘I feel like the disease is less risky than exposing her or my son to the chemicals and preservatives and the vaccines[.]’); See also Sara Berg, *What Doctors Wish Patients Knew about How Well COVID-19 Vaccines Work*, ama-assn.org (Oct 21, 2022), https://www.ama-assn.org/delivering-care/public-health/what-doctors-wish-patients-knew-about-how-well-covid-19-vaccines-work (‘“But there’s not a single infectious disease, actually, that has reached that status without a vaccine through the history of time,” she added’.) (hereinafter ‘AMA’).

41 Joanna K. Sax & Neal M. Doran, *Ambiguity and Consumer Perceptions of Risk in Various Areas of Biotechnology*, J. of  Consumer  Pol’y 47, 55 (2018) (‘As hypothesized, we found that participants who showed an initial aversion to ambiguous information were significantly more likely to choose the response options that indicated a high assignment of risk, even when alternative choices had a high benefit or a likelihood of low risk’.).

42 See generally, Joanna K. Sax, *COVID-19 Vaccine Hesitancy and (Mis)perception of Risk*, 48 Am. J. of L. and  Med. 54, 69 (2022).

43 Ana Santos Rutschman, *Social Media Self-Regulation and the Rise of Vaccine Misinformation*, 4 U. Pa. J. L. & Innov. 25, 28 (2022).

44 SB-277 Public health: vaccinations (2015–16), https://leginfo.legislature.ca.gov/faces/billTextClient.xhtml?bill_id=201520160SB277 (‘This bill would eliminate the exemption from existing specified immunization requirements based upon personal beliefs[.]’); see also, Michelle M. Mello et al., *Effectiveness of Vaccination Mandates in Improving Uptake of COVID-19 Vaccines in the USA*, 400 Lancet 535, 535 (2002) (‘The stringency and enforcement of school-entry mandates matter. State that have eliminated personal belief or religious exemptions (while maintaining medical exemptions) have lower exemption rates and higher vaccination rates. Further, outbreaks of vaccine-preventable disease have disproportionately occurred in areas with higher exemption rates. Such evidence has prompted California, Connecticut, Maine, Mississippi, New York, Washington, and West Virginia to remove religious or personal-belief exemptions from some or all vaccination requirements. Likewise, states that impose burdensome procedural requirements to obtain exemptions – such as counseling, annual reapplication, notarization, or clergy attestation – have lower exemption rates and lower risk of disease outbreak’. [internal citations omitted]).

45 See, e.g. Rutschman, *supra* note 44, at 31–2 (‘In recent years, however, wavering trust in vaccines has been deemed one of the most significant contributing factors towards declining rates of vaccination, particularly across the Western world[]’ and citations therein).

46 Sax, *supra* note 43, at 70.

47
*See* Centers for Disease Control and Prevention*, Estimates of vaccine hesitancy for COVID-19*, https://data.cdc.gov/stories/s/Vaccine-Hesitancy-for-COVID-19/cnd2-a6zw/ (accessed May 1, 2023).

48 Grace Sparks et al., *KFF COVID-19 Vaccine Monitor: September 2022*, (Sept. 30, 2022), https://www.kff.org/coronavirus-covid-19/poll-finding/kff-covid-19-vaccine-monitor-september-2022/ (‘Overall adult vaccination rates have been relatively steady over the past year. The latest COVID-19 Vaccine Monitor finds that nearly eight in ten adults (77%) say they have gotten at least one dose of a COVID-19 vaccine, including about half who say they are fully vaccinated and also received at least one booster dose (47%), about a quarter who have been fully vaccinated but have not gotten a booster (26%), and a small share who are partially vaccinated (3%). Twenty-three percent remain unvaccinated, the vast majority of whom say they will “definitely not” get the COVID-19 vaccine (88% of unvaccinated, or 21% of all adults’. [Compare with] ‘About one in five (19%) parents of children ages 6 months through 4 years old say their child has gotten vaccinated for COVID-19, up from 7% in July. The September Monitor survey finds about half (53%) of parents of children in this age range say they will “definitely not” get their child vaccinated for COVID-19. Reported vaccine uptake among children ages 5–11 and teenagers ages 12–17 has slowed in recent months. Almost half of parents of kids ages 5–11 now report their child has gotten vaccinated (46%), as do 62% of parents of teens ages 12–17’.).

49 Lunna Lopes et al., *KFF COVID-19 Vaccine Monitor: July 2022*, (July 26, 2022), https://www.kff.org/coronavirus-covid-19/poll-finding/kff-covid-19-vaccine-monitor-july-2022/.

50
*Id*.

51
*Id*. (This portion of the KFF survey related to parents of children between ages 6 months and 4 years but is used herein as examples of vaccine hesitant parents).

52 Blaisdell, *supra* note 40, at 479.

53 Pfizer, *Harnessing the Potential of mRNA*, https://www.pfizer.com/science/innovation/mrna-technology (accessed Jul. 31, 2023) (‘mRNA—or messenger RNA—is a molecule that contains the instructions or recipe that directs the cells to make a protein using its natural machinery. To enter cells smoothly, mRNA travels within a protective bubble called a Lipid Nanoparticle. Once inside, our cells read the mRNA as a set of instructions, building proteins that match up with parts of the pathogen called antigens’.).

54 5 U.S.C. § 551 *et seq*. (1946), as amended.

55 Food and Drug Administration, *Vaccines*, (last updated Feb. 8, 2023), https://www.fda.gov/vaccines-blood-biologics/vaccines (‘Vaccines, as with all products regulated by FDA, undergo a rigorous review of laboratory and clinical data to ensure the safety, efficacy, purity and potency of these products. Vaccines approved for marketing may also be required to undergo additional studies to further evaluate the vaccine and often to address specific questions about the vaccine’s safety, effectiveness or possible side effects’.); Centers for Disease Control and Prevention, *Role of the Advisory Committee on Immunization Practices in CDC’s Vaccine Recommendations*, (last reviewed Oct. 8, 2020), https://www.cdc.gov/vaccines/acip/committee/role-vaccine-recommendations.html# (‘The Centers for Disease Control and Prevention (CDC) sets the U.S. adult and childhood immunization schedules based on recommendations from the Advisory Committee on Immunization Practices (ACIP)’.).

56 5 U.S.C. § 2(a) (1972) (‘The Congress finds that there are numerous committees, boards, commissions, councils, and similar groups which have been established to advise officers and agencies in the executive branch of the Federal Government and that they are frequently a useful and beneficial means of furnishing expert advice, ideas, and diverse opinions to the Federal Government’.).

57 See FDA, *Advisory Committee Laws, Regulations and Guidance*, (last updated Mar. 27, 2018), https://www.fda.gov/advisory-committees/about-advisory-committees/advisory-committee-laws-regulations-and-guidance.

58 2001 Federal Advisory Committee Act (FACA) Final Rule of 2001 at § 102–3.150, (last reviewed Jan. 7, 2022), https://www.gsa.gov/policy-regulations/policy/federal-advisory-committee-management/legislation-and-regulations/faca-final-rule-2001.

59 COVID-19 Vaccine Docket, *supra* note 11.

60
*Id.*

61 Food and Drug Administration, 170th Meeting of the Vaccines and Related Biological Products Advisory Committee (Oct. 26, 2021), https://www.youtube.com/watch?v=laaL0_xKmmA

62
*Id.* at approximately 7:40.

63
*Id.* at approximately 7:48–7:50 (18 voting members total: 17 voted yes and 1 abstained). Risk determination by scientific experts is not without its criticism. Scientists are influenced by biases and other external factors. Sometimes, new information becomes available that changes the risk assessment. See, e.g. Int’l Dairy Foods Ass’n v. Amestoy, 92 F. 3d 67, 76–77 (2nd Cir. 1996) (dissent) (‘Furthermore, there are many possible reasons why a government agency might fail to find real health risks, including inadequate time and budget for testing, insufficient advancement of scientific techniques, insufficiently large sampling populations, pressures from industry, and simple human error’.).

64 Food and Drug Administration, *FDA Authorizes Pfizer-BioNTech COVID-19 Vaccine for Emergency Use in Children 5 through 11 Years of Age*, (content current as of Oct. 29, 2021), https://www.fda.gov/news-events/press-announcements/fda-authorizes-pfizer-biontech-covid-19-vaccine-emergency-use-children-5-through-11-years-age (‘Today, the U.S. Food and Drug Administration authorized the emergency use of the Pfizer-BioNTech COVID-19 Vaccine for the prevention of COVID-19 to include children 5 through 11 years of age. The authorization was based on the FDA’s thorough and transparent evaluation of the data that included input from independent advisory committee experts who overwhelmingly voted in favor of making the vaccine available to children in this age group’.).

65
*Cf*. MaryBeth Musumeci and Jennifer Kates, *Key Questions About COVID-19 Mandates,* (Apr. 7, 2021), https://www.kff.org/coronavirus-covid-19/issue-brief/key-questions-about-covid-19-vaccine-mandates/ (‘The Federal government’s authority to institute a general vaccine mandate is unclear, and has not yet been tested in the courts, though it is likely limited at best’) (original in bold); see also Jacobson v. Massachusetts, 197 U.S. 11 (1905) (upholding a state vaccine mandate).

66 5 U.S.C. § 553 (2012). The notice of the meeting of the VRBPAC is not rulemaking pursuant to 5 U.S.C. § 553; however, a discussion of the role of public comments is applicable here. See also, James V. DeLong, *Informal Rulemaking and the Integration of Law and Policy*, 65 Va. L. Rev. 257, 257–8 (1979) (discussing notice and comment as part of informal rulemaking).

67 Edward L. Rubin & Joanna K. Sax, *Administrative Guidance and Genetically Modified Food*, 60 Ariz. L. Rev. 539, 581 (2018).

68 One area in which the debate is highlighted is in an agency’s decision to use guidance instead of notice and comment rulemaking. The scholarly debate in this area is instructive for thinking about the utility of public comments for the VRBPAC meeting. See Nina A. Mendelson, *Regulatory Beneficiaries and Informal Agency Policymaking*, 92 Cornell L. Rev. 397, 402 (2007) (describing ‘regulatory beneficiaries’ as people who benefit indirectly from agency action, such as ‘employees who expect healthier workplaces, consumers who seek safer products, and those who hope to enjoy and benefit from a cleaner environment’.); see also, Rubin & Sax, *supra* note 68, at 581, fns 215, 216 (2018) (‘While some scholars view notice and comment rulemaking as a great innovation, others question its effectiveness’.).

69 See U.S. General Services Administration, *The Federal Advisory Committee Act (FACA) Brochure*, (last reviewed Feb. 26, 2019), https://www.gsa.gov/policy-regulations/policy/federal-advisory-committee-management/advice-and-guidance/the-federal-advisory-committee-act-faca-brochure (‘Under the provisions of the Federal Advisory Committee Act, federal agencies sponsoring advisory committees must:  • Arrange meetings that are reasonably accessible and at convenient locations and times;  • Publish adequate advance notice of meetings in the *Federal Register*;  • Open advisory committee meetings to the public (with some exceptions-see the section on   “Government in the Sunshine Act” below);  • Make available for public inspection, subject to the Freedom of Information Act, papers and records,   including detailed minutes of each meeting; and  • Maintain records of expenditures.’).

70 For an example, see Dept. of Transportation Federal Aviation Administration, 87 Fed. Reg. 5389 (Feb. 1, 2022) (‘The FAA received a comment from the Air Line Pilots Association, International (ALPA), who supported the NPRM without change’.); Dept of the Interior, National Park Service, 87 Fed. Reg. 5402, 5403 (Feb. 1, 2022) (‘The NPS received four comments on the proposed rule. Below are summaries of the pertinent issues raised in the comments and responses from the NPS. After considering the public comments and after additional review, the NPS did not make any changes to the proposed rule’.); see also, Rubin & Sax, *supra* note 68, at 584, fn 225.

71 See Rubin & Sax, *supra* note 68, at 584; see, e.g. Dept of Commerce, National Oceanic and Atmospheric Administration, 87 Fed. Reg. 5405, 5406 (Feb 1, 2022) (adopting a final rule regarding final small-mesh multispaces specifications and stating that ‘NMFS received no comments from the public’.). Presumably, the regulated parties are in close contact with NOAA and the final rule refers to the ‘New England Fishery Management Council[.]’

72 See Hoctor v. US Dept. of Agriculture, 82 F.3d 165, 171 (7th Cir 1986) (addressing whether notice and comment was required for what an agency deemed to be an interpretative rule, Judge Posner responded to the Department lawyer’s argument that ‘if notice and comment route had been followed in this case the Department would have received thousands of comments[]’ by saying that ‘The greater the public interest in a rule, the greater reason to allow the public to participate in its formation’.).

73 The number 130.47 K is abbreviated on the public docket. It is unclear why a discrepancy exists as to the number of comments received as compared with the total number of related comments.

74 The decision to analyze ~1500 comments was based on several studies analyzing comments or posts on various social media and web-based platforms. See, e.g. Jeanine P.D. Guidry, Kellie Caryle, Marcus Messner & Yan Jin, *On Pins and Needles: How Vaccines are Portrayed on Pinterest*, 33 Vaccine 5051, 5052 (2015); Esther Ainley, Cara Witwicki, Amy Tallent & Chris Graham, *Using Twitter Comments to Understand People’s Experiences of UK Health Care During the COVID-19 Pandemic: Thematic and Sentiment Analysis*, 23 J. Med. Internet  Res. e31101 (2021); Gautam Kishore Shahi, Anne Dirkson & Tim A. Maychrzak, *An Exploratory Study of COVID-19 Misinformation on Twitter*, 22 Online  Social  Media  Networks and  Media 100,104, 7 (2021). The use of the API key to pull the comments was needed because regulations.gov did not post all the comments. This is apparently due to a technical issue with the website. In most scenarios, regulations.gov will post all comments, but the 137,000 comments proved too many for regulations.gov Importantly, the comments can be accessed using an API key.

75 Regulations.gov does not have the capacity to post more than 500 comments submitted per day. The first author submitted an FOIA request to obtain all the comments because not all comments were publicly available on regulations.gov. Through conversations with a representative of the FDA, the first author withdrew the FOIA request because access to the comments was provided through an API key. The first author and the research assistants received API keys to access the comments.

76 Full list of words: (Mandate(s)(d)/mandating/required/force(s)(d)/forcibly/coerced/mandatory/dead/ danger /dangerous/death/obligat(e)(ed), deadly, died, kill(ed), require(e)(ed)(ing); dangers and deaths were included in reconciliation); see Slovic 2014, *supra* note 7, at 977 (‘The significance of this finding for the affect heuristic was not realized until a study by Alhakami and Slovic found that the inverse relationship between perceived risk and perceived benefit of an activity (e.g., using pesticide) was linked to the strength or positive or negative affect associated with that activity as measure by the rating of the activity on bipolar scales such as *good/bad, nice/awful, dreaded/not* dreaded, and so forth.’); Slovic & Peters 2006, *supra* note 27, at 324 (‘When the consequences of an action or event carry strong affective meaning, as is the case with a lottery jackpot or a cancer, the probability of such consequences often carries too little weight’.); Slovic (2007), *supra* note 24, at 1334 (2007) (‘We do not just see “a house”: We see a *handsome* house, an *ugly* house, or a *pretentious* house’’.) Comments may not just see a vaccine, but see a *mandated* vaccine, *forced* vaccine, for example.

77 Blaisdell, *supra* note 40, at 483.

78 Menelaos Konstantinidis Lisa W. Le, & Xin Gao, *An empirical comparative assessment of inter-rater agreement of binary outcomes and multiple raters*, 14 Symmetry 262, 262–70 (2022).

79 All comments are reproduced as found using the API key. Coders did not correct for syntax or other potential ‘cut & paste’ issues so that the full original comment was analyzed.

80 Vanessa Piechotta et al., *Safety and Effectiveness of Vaccines Against COVID-19 in Children Aged 5–11 years: a Systematic Review and Meta-Analysis*, 7 The  Lancet  Child & Adolescent  Health 379, 379 (2023).

81 Projects are underway to understand long-COVID. See National Institutes of Health, *Long COVID*, https://covid19.nih.gov/covid-19-topics/long-covid (accessed May 1, 2023) (‘Children and teenagers can get long COVID, whether they had COVID-19 symptoms or not’.); National Institutes of Health, *RECOVER: Researching COVID to Enhance Recovery*  https://recovercovid.org/ (accessed July 31, 2023) (hereinafter ‘RECOVER’).

82 RECOVER, *supra* note 82.

83 AMA, *supra* note 41.

84 Cavatorta & Schröder, *supra* note 39, at 72.

85 The risk assessment also included clinical trials in pediatric populations.

86 Sax, *supra* note 43, at 86 (‘This Article posits that unless we align individual perceptions of risk with evidence-based assessment of risk, our current strategies for legal policy implementation will be less and less effective. In other words, this Article aims to address the root cause of such hesitancy so legal policies have the best chance to obtain the desired behavior. The specific of COVID-19 vaccine hesitancy and policy lend itself to the root cause analysis, especially due to the missteps in the handling of the pandemic’.).

87 Executive Order 12866, Regulatory Planning and Review, 58 Fed. Reg. 12866, Monday Oct 4, 1993 (‘In deciding whether and how to regulate, agencies should assess all costs and benefits of available regulatory alternative, including the alternatives of not regulating’.); Matthew D. Adler & Eric A. Posner, *Rethinking Cost–Benefit Analysis*, 109 Yale L. J. 165, 167 (1999) (‘Government agencies now routinely use CBA. This was not always the case. Before the 1980s, agencies did not systematically rely on CBA when evaluating regulations and other projects. But executive orders issued by the Reagan and Clinton administrations have since made the use of CBA by agencies common, and Congress has enacted numerous statutes requiring agencies to perform cost–benefit analyses [internal footnotes omitted]’.).

88 See Mello, *supra* note 45, at 535 (2022) (‘Mandates, however, are controversial in many countries. Austria’s proposed mandate for adults, for example, provoked mass protests. Some objectors argue mandates represent encroachment on individual liberty. Some other objectors maintain that mandates will not be an effective policy for COVID-19 because many individuals will seek to evade them, and mandates might erode support for other public health measures such as mask wearing’.).

89
*Cf*., Slovic 2007, *supra* note 24, at 1334 (‘We do not just see “a house”: We see a *handsome* house, an *ugly* house, or a *pretentious* house’.). Comments may not just see a vaccine, but see a *mandated* vaccine, *forced* vaccine, for example.

90
*Cf*., Kenny Skagerlund, Mattias Forsblad, Paul Slovic & Daniel Västfjäll, *The Affect Heuristic and Risk Perception – Stability Across Elicitation Methods and Individual Cognitive Abilities*, 11 Frontiers in  Psychology 1, 1–3 (2020) (describing numerous empirical studies evaluating the role of the affect heuristic in risk perception).

91 Slovic 2014, *supra* note 7, at 990.

92 See, e.g. Dan M. Kahan, Donald Braman, Paul Slovic, John Gastil, and Geoffrey Cohen, *Cultural Cognition and the Risks and Benefits of Nanotechnology*, 4 Nature  Nanotechnology 87, 87–88 (2008).

93 P. Marijn Poortvliet, Martijn Duineveld & Kai Purnhagen, *Performativity in Action: How Risk Communication Interacts in Risk Regulation*, 1 Eur. J. Risk  Regulation 213, 213–215, 217 (2016).

94 James V. DeLong, *Informal Rulemaking and the Integration of Law and Policy*, 65 Va. L. Rev. 257, 302 (1979) (discussing efforts to infuse democratic values into agency action).

95 See, e.g. Federal Aviation Administration (FAA), *Airworthiness Directives; Airbus Helicopters*, A Rule Issued by the FAA, (Oct. 11, 2022), https://www.federalregister.gov/documents/2022/10/11/2022-21949/airworthiness-directives-airbus-helicopters (‘The FAA received no comments on the NPRM or on the determination of the costs’.).

96 E. Donald Elliott, *Re-Inventing Rulemaking*, 41 Duke L. J. 1490. 1492–3 (1992) (internal citations omitted).

97 Rubin & Sax, *supra* note 68, at 580 (‘The question that the reviewing court might ask when a document that the agency promulgates as guidance is challenged for failure to comply with section 553, is whether notice and comment rulemaking would serve a useful purpose in the promulgation of the document in question’.)

98 Edward Rubin, *It’s Time to Make the Administrative Procedure Act Administrative*, 89 Cornell L. Rev. 95, 101 (2003).

99
*Id*. at 102.

100
*Id*. at 102 (2003); see, e.g. Federal Reserve System, *Debit Card Interchange Fees and Routing*, Final Rule (Oct. 11, 2022), https://www.federalregister.gov/documents/2022/10/11/2022-21838/debit-card-interchange-fees-and-routing (stating that the Board received slightly more than 2750 comments, ~1700 were from debit card issuers).

101 Or, it could be a combination of comments organized by a public interest group and individual commenters. See Rubin, *supra* note 99, at 115 (‘one comment may come from a public interest group working exclusively in the field that the proposed regulation covers, and thus would represent the considered judgments of many leading experts in that field; another may be the causal ravings of a deranged or dyspeptic amateur’.). In our study, none of the 1500 comments pulled at random appeared to be from an organization, rather commenters were identified by individual names or posted anonymously.

102 Some commenters expressed concerns about myocarditis as a possible side effect of the vaccine. For example, comment ID 6198 stated: ‘As a Board Certified Child Psychiatrist I am gravely concerned about vaccinating children between the ages of 5–11 with the Pfizer vaccine. The grave concern arises from the known side effects from the vaccine. This vaccine is still experimental and the potential risks far outweigh the benefits. Specifically myocarditis in young males is very serious and this vaccine needs much more time to study before it is mandated to children. I am pro vaccine as long as it is tested adequately for safety’. COVID-19 Vaccine Docket, *supra* note 11. Myocarditis was a known issue to the advisory committee and was discussed during the open meeting. Since then, the data tell us that an infection with COVID-19 poses a higher risk for myocarditis as compared with the risk of myocarditis from the COVID-19 vaccine. See American Heart Association News, *COVID-19 infection poses higher risk for Myocarditis than Vaccines*, (Aug. 22, 2022), https://www.heart.org/en/news/2022/08/22/covid-19-infection-poses-higher-risk-for-myocarditis-than-vaccines (‘The overall risk of myocarditis – inflammation of the heart muscle – is substantially higher immediately after being infected with COVID-19 than it is in the weeks following vaccination for the coronavirus, a large new study in England shows’.).

103 COVID-19 Vaccine Docket, *supra* note 11.

104 It is possible that the agency views itself differently than how the public views the agency. For example, the agency may view itself as a mechanism to provide education, but it is unclear if the public is truly educated. See, e.g. Margaret A. Hamburg & Joshua M. Sharfstein, *The FDA as a Public Health Agency*, 360 New  Eng. J. Med. 2493, 2494 (2009) (‘The FDA’s job is to minimize risks through education, regulation, and enforcement. To be credible in all these tasks, the agency must communicate frequently and clearly about risks and benefits — and about what organizations and individuals can do to minimize risk. When, like the FDA, Americans must make choices about medication, devices, foods, or nutrition in the absence of perfect information, the FDA cannot delay in providing reasonable guidance — guidance that informs rather than causes unnecessary anxiety’.). Given the misperception of risk of members of the public toward the COVID-19 vaccine, it begs the question as to whether the FDA’s goal should be to minimize risk or to educate about risk.

105 Food and Drug Administration, *What We Do*, https://www.fda.gov/about-fda/what-we-do (accessed July 31, 2023) (‘The Food and Drug Administration is responsible for protecting the public health by ensuring the safety, efficacy, security of human and veterinary drugs, biological products, and medical devices; and by ensuring the safety of our nation’s food supply, cosmetics, and products that emit radiation’.).

106 The Federal Register and the public comment process is not inherently obvious. The Federal Register posts the daily operations of the government and one wonders how many typical individuals read the Federal Register on a daily basis. See National Archives, *About the Federal Register*, (last updated Aug. 8, 2018), https://www.archives.gov/federal-register/the-federal-register/about.html (‘**Who uses the *Federal Register*?** Anyone: • who needs to know about the day-to-day operations of the Federal Government • whose business is regulated by a Federal agency • who is an attorney practicing before a regulatory agency • who attends public hearings or meetings or applies for grants • who is concerned with Government actions that affect the environment, health care, financial services, exports, education, or other major public policy issues).’

107 Admittedly, this poses potentially significant barriers to public participation, so this suggestion needs to be carefully considered and adjusted.

108 A large body of literature discusses how affect, for example, impacts risk perception. A smaller body of literature addresses using affect, for example, as an intervention strategy. See, e.g. Wändi Bruine de Bruin et al., *Promoting Protection Against a Threat That Evokes Positive Affect: The Case of Heat Waves in the United Kingdom*, 22 J. Experimental  Psychology: Applied 261, 261–2, 268 (2016) (describing an experiment using availability heuristic and affect heuristic as an intervention to change risk perception regarding hot summers). Similarly, a smaller body of literature discusses how resolving ambiguity, or learning about ambiguity aversion, can be used as an intervention strategy. See, e.g. Ruonan Jia et al., *Learning about the Ellsberg Paradox Reduces, but does not Abolish, Ambiguity Aversion*, PLos  One 1, 1 (Mar. 4, 2020) (‘Participants who learned about the Ellsberg paradox were more tolerant of ambiguity, yet ambiguity aversion was not completely abolished. At the same time, these participants also exhibited reduced aversion to risk, suggesting inappropriate generalization of learning to an irrelevant decision domain. Our results highlight the challenge for behavioral interventions: generating a strong, yet specific, behavioral change’.).

109 A possible framework is a Just-in-Time intervention. See Inbal Nahum-Shani et al., *Just-in-Time Adaptive Interventions (JITAIs) in Mobile Health: Key Components and Design Principles for Ingoing Behavior Support*, 52 Ann. Behav. Med. 446 (2018).

110 Because this was an Advisory Committee meeting, the APA does not require the written response to comments that is done in response to rulemaking. See US v. Nova Scotia Food Products, Corp., 568 F. 2d 240, 253 (2nd Cir. 1977) (explaining that a general concise statement was inadequate to respond to a specific comment).

111 In the United States, from Jan. 2020 through Oct. 12, 2022, the CDC reports 1490 pediatric deaths (ages 0–18) involving COVID-19. Centers for Disease Control, *Provisional COVID-19 Deaths: Focus on Ages 0–18 Years*, (last updated Apr. 26, 2023), https://data.cdc.gov/NCHS/Provisional-COVID-19-Deaths-Focus-on-Ages-0-18-Yea/nr4s-juj3. While pediatric deaths involving COVID-19 are smaller as compared with adult deaths involving COVID-19, which reached over 1 million as of Oct. 2022, any death is tragic; see also, Erika Edwards, *Children are Hospitalized with Covid at record numbers*, nbcnews.com, (Jan. 3, 2022 at 3:38 pm, updated Jan. 5, 2022 at 10:10 am), https://www.nbcnews.com/health/health-news/covid-warning-symptoms-children-kids-hospitalized-record-numbers-rcna10741.

112 Kristie E. N. Clarke, *Seroprevalence of Infection-Induced SARS-CoV-2 Antibodies – United States, September 2021–February 2022,*  cdc.gov (Apr. 29, 2022) https://www.cdc.gov/mmwr/volumes/71/wr/mm7117e3.htm; Erika Edwards, *CDC Report Finds 75 Percent of Children and Teens had Covid by February,*  nbcnews.com (Apr. 11, 2022 at 10:55 am, updated Apr. 26, 2022 at 2:56 pm), https://www.nbcnews.com/health/health-news/cdc-says-75-percent-children-covid-february-rcna26029 (‘The increase in Covid was most significant in kids: from 44.2 percent in December to 75.2 percent in February among children ages 11 and younger, the CDC report found. The percentages were almost identical for kids ages 12 to 17’.); Victor Chernozhukov, Hiroyuki Kasahara and Paul Schrimpf, *The Association of Opening K-12 Schools with the Spread of COVID-19 in the United States: County-level Panel Data Analysis*, 118 Proceedings of the  Nat’l  Acad. of  Sci. 1, 1 (2021) (‘The estimates indicate that fully opening K-12 schools with in-person learning is associated with a 5 (SE+2) percentage points increase in the growth rate of cases’.)

113 National Institutes of Health, *Understanding the Long-Term Effects of COVID-19 in Children* (last updated Dec. 23, 2021), https://covid19.nih.gov/news-and-stories/understanding-long-term-effects-covid-19-children (‘And COVID-19 may have subtler long-term effects on children that scientists do not yet know’.).

114 The National Institutes of Health is studying children to understand the long-term effects of COVID-19. *Id*.

115 Gwenaëlle Douaud et al., *SARS-CoV-2 is Associated with Changes in Brain Structure in UK Biobank*, Nature, accelerated publication (2022) (finding both imaging and cognitive longitudinal defects in patients who were infected with the virus that causes COVID-19 and adding that it remains to be understood if these brain effects will persist in the long term).

116 See Yanyao Du et al., *Two-Year Follow-up of Brain Structural Changes in Patients Who Recovered from COVID-19: A Prospective Study*, 319 Psychiatry  Res. 114969, 114969–70 (and references therein).

117
*Id*. at 114974.

118 Annika V. Ballering, Sander K. R. van Zon, Tim C. olde Hartman & Judith G.M. Rosmalen, *Persistence of Somatic Symptoms After COVID-19 in the Netherlands: An Observational Cohort Study*, 400 Lancet 452, 452 (2022) (finding ‘[p]ersistent symptoms in COVID-19-positive participants at 90–150 days after COVID-19 compared with before COVID-19 and compared with matched controls included chest pain, difficulties with breathing, pain when breathing, painful muscles, ageusia or ansomia, tingling extremities, lump in throat, feeling hot and cold alternatively, heavy arms or legs, and general tiredness’.); Amir Tajbakhsh et al., *COVID-19 and Cardia Injury: Clinical Manifestations, Biomarkers, Mechanisms, Diagnosis, Treatment, and Follow Up*, 19 Expert  Rev. of  Anti-Infective  Therapy 345, 345–6 (2021) (reviewing the literature on the effect of COVID-19 on the cardiovascular system).

119 Du et al., *supra* note 117 at 114972.

120 Tom Shimabukuro, *Update on myocarditis following mRNA COVID-19 vaccination*, Centers for Disease Control, (dated June 23, 2022), https://www.cdc.gov/vaccines/acip/meetings/downloads/slides-2022-06-22-23/03-covid-shimabukuro-508.pdf (presenting information about following and monitoring information about myocarditis as it relates to COVID-19 vaccination).

121 The vaccine does not necessarily prevent infection; rather, it is effective at preventing serious illness. Thus, it is still possible that a vaccinated individual will get COVID-19 and experience the same long-term effects as an unvaccinated individual. It is also possible that a vaccinated individual will get COVID-19 and experience less long-term effects than an unvaccinated individual who gets COVID-19.

122 A separate and distinct question is whether in informal rulemaking, whether commenters read the final rule and the agency’s response to a specific comment. For regulated entities, the answer is probably yes because they must comply with the final rule. If a commenter is not a regulated entity, it would be interesting to learn whether they read the final rule and the agency’s response to their comment.

123 In addition, the severity of the coronavirus strain circulating at that time may also impact perceptions of risk related to a vaccine. Put differently, omicron’s seemingly milder symptoms may impact how individuals perceive the risk of the vaccine as compared with the risk of the disease.

124 See, e.g. Cavatorta & Schröder, *supra* note 39 at 92, Table 6.

